# *PyPhase* – a Python package for X-ray phase imaging

**DOI:** 10.1107/S1600577521004951

**Published:** 2021-06-25

**Authors:** Max Langer, Yuhe Zhang, Diogo Figueirinhas, Jean-Baptiste Forien, Kannara Mom, Claire Mouton, Rajmund Mokso, Pablo Villanueva-Perez

**Affiliations:** a Univ Lyon, INSA Lyon, Université Claude Bernard Lyon 1, UJM-Saint Etienne, CNRS, Inserm, CREATIS UMR 5220, U1206, F-69621 Villeurbanne, France; b Division of Synchrotron Radiation Research and NanoLund, Department of Physics, Lund University, SE-221 00 Lund, Sweden; c Division of Packaging Logistics, Faculty of Engineering, Lund University, SE-22100 Lund, Sweden; d MAX IV Laboratory, Lund University, SE-22100 Lund, Sweden; e Lawrence Livermore National Laboratory, Livermore, CA 94550, USA; f Division of Solid Mechanics, Faculty of Engineering, Lund University, SE-22100 Lund, Sweden

**Keywords:** X-ray imaging, phase contrast, holography, phase retrieval, tomography

## Abstract

X-ray propagation-based imaging techniques are well established at synchrotron radiation and laboratory sources. However, most reconstruction algorithms for such image modalities, also known as phase-retrieval algorithms, have been developed specifically for one instrument by and for experts, making the development and diffusion of such techniques difficult. Here, *PyPhase*, a free and open-source package for propagation-based near-field phase reconstructions, which is distributed under the CeCILL license, is presented. *PyPhase* implements some of the most popular phase-retrieval algorithms in a highly modular framework supporting its deployment on large-scale computing facilities. This makes the integration, the development of new phase-retrieval algorithms, and the deployment on different computing infrastructures straightforward. Its capabilities and simplicity are presented by application to data acquired at the synchrotron source MAX IV (Lund, Sweden).

## Abstract 2

*PyPhase* is a free and open-source Python package for propagation-based near-field phase reconstructions, which implements some of the most popular phase-retrieval algorithms in a highly modular framework supporting the deployment on large-scale computing facilities. This makes integration, development of new phase-retrieval algorithms, and the deployment on different computing infrastructures straightforward.

## 1. Introduction   

The unique penetration power and short wavelength of X-rays make them an excellent probe to explore nature in a non-destructive manner down to the atomic scale. However, the high penetration power can lead to low contrast in imaging, especially for microscopic samples when exploiting attenuation contrast. Propagation-based techniques can enhance the contrast and sensitivity by exploiting phase contrast. X-ray propagation-based phase-contrast imaging has seen a large increase in interest and development since its origins 25 years ago (Snigirev *et al.*, 1995 ▸; Wilkins *et al.*, 1996 ▸). These techniques have seen widespread use, particularly for soft tissues, and for weakly contrasting structures in a dense matrix. With the use of phase-retrieval algorithms together with computed tomography, the complex refractive index distribution in the imaged object can be reconstructed in 3D.

**Figure 1:**
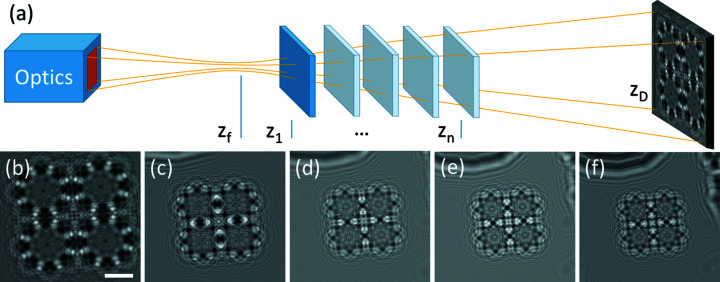
Phase-contrast imaging. (*a*) Schematic of the experimental setup for high-resolution phase-contrast imaging. An X-ray beam is focused using reflective (Kirkpatrick–Baez) optics. The sample is placed at different positions *z*_*n*_ relative to the focus (*z*_f_) and detector (*z*_D_) positions for different amounts of magnification and consequently different effective propagation distances. (*b*)–(*f*) Phase-contrast images acquired at sample positions progressively further from the focus (and thus closer to the detector) showing the varying degree of magnification and phase contrast (acquired at NanoMAX, MAX IV, Lund, Sweden). The scale bar is shown for the highest-resolution image and corresponds to 2.5 µm.

Near-field propagation-based techniques record images that can be described as the intensity of the Fresnel transform of the exit wave as a function of energy, propagation distance, and length scale. The first images recorded with these approaches had resolutions not better than the optical domain. More recently, the advent of X-ray optics has offered the opportunity to image at higher resolutions than conventional light microscopy (Mokso *et al.*, 2007 ▸). Figure 1 ▸(*a*) depicts a phase-contrast experimental setup using X-ray optics for high-resolution imaging. The images recorded by such experimental setups approach the holographic regime. In this regime, the direct interpretation of the images becomes more and more difficult with increasing resolution. Examples of holographic images of a test pattern, from higher to lower magnification, are shown in Figs. 1 ▸(*b*)–1(*f*). The images were acquired at beamline NanoMAX at the MAX IV synchrotron (Lund, Sweden). X-rays were focused using Kirkpatrick–Baez reflective optics (Johansson *et al.*, 2018 ▸). The X-ray energy was set to 13 keV, yielding a focal spot of 70 nm. A CRYCAM microsystem camera from Crytur was used for detection, using a LuAG(Ce) scintillator, 10× visible light optics, and an Andor Zyla 4.2 Plus CCD camera yielding a detector pixel size of 650 nm. The detector was positioned at *z*_D_ = 1.12 m from the focal spot. For the highest-resolution image, the sample was placed at *z*_1_ = 10.1 mm yielding a geometric magnification factor of *M* = 110.9. The effective pixel size was measured to be 6 nm. Reconstructing the phase (and possibly the amplitude) of the exit wave using phase retrieval becomes a mandatory step for exploiting such images.

The phase-retrieval step has proved to be a persistent challenge, as no general algorithm currently exists. The practitioner is instead left to pick from a large range of algorithms that have been proposed in the literature. Initially, phase-retrieval algorithms were based on linearizations of the Fresnel integral, *e.g.* with respect to the exit wave itself (Gabor, 1948 ▸). However, this solution is not applicable to all the imaging conditions and samples.

It is useful to classify propagation-based techniques into two regimes to understand their applicability and challenges. These two regimes are divided with respect to the relative propagation distance. Relatively short propagation distances with respect to the imaging length scale and wavelength yield what is sometimes called the *edge-enhancement* regime. This translates to a Fresnel number *F* = *a* ^2^/λ*L*, with *a* the size of the imaged feature, λ the wavelength, and *L* the propagation distance, corresponding to the smallest feature in the image that is *F* ≃ 1. In this regime, phase contrast mainly enhances interfaces between materials in the sample with a visible diffraction fringe. Relatively long distances, corresponding to 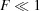 but still within the validity of Fresnel diffraction, give rise to what is sometimes known as the *holographic* regime, where the diffraction fringes dominate the contrast and are spread over a long distance in the object.

In the edge-enhancement regime, algorithms based on assumptions on the imaged object, *e.g.* homogeneity of the constituent materials (Paganin *et al.*, 2002 ▸; Langer *et al.*, 2010 ▸), have seen widespread use. Such algorithms are simple and require only a single frame. These algorithms have been very successful for nearly homogeneous objects with fine variations in a dense matrix, but limitations of use remain for many types of samples due to violation of the homogeneity assumption.

More recently, algorithms taking into account the non-linearity of the problem have been proposed, which can be applied to the edge-enhancement and holographic regimes. Some take directly into account the specificity of the Fresnel framework (Davidoiu *et al.*, 2011 ▸), whereas others borrow from the rich literature on reconstruction algorithms from coherent diffraction imaging and crystallography, based on projection onto sets type algorithms (Gerchberg & Saxton, 1972 ▸; Fienup, 1980 ▸; Elser, 2003 ▸).

Recent developments in phase reconstruction consider the problem in 3D, either through regularization using prior information (Langer *et al.*, 2012*a* ▸, 2014 ▸; Kostenko *et al.*, 2013 ▸), filtering (Brun *et al.*, 2019 ▸), iterative schemes in the object domain (Ruhlandt & Salditt, 2016 ▸), or in both object and projection domain (Ruhlandt *et al.*, 2014 ▸). This allows, for example, the use of constraints in the object domain in the phase-retrieval procedure, or improve the computational efficiency.

Although there is a plethora of literature regarding phase-retrieval algorithms, access to implementations of those algorithms is relatively closed, with a few exceptions (Lohse *et al.*, 2020 ▸; Weitkamp *et al.*, 2011 ▸; Gürsoy *et al.*, 2014 ▸). To address this limitation, we present a complete Python phase-retrieval package for X-ray phase-contrast imaging, named *PyPhase*. PyPhase is a fully open-source and modular package that relies solely on free and open-source tools.

## 2. Organization of the package   

With *PyPhase*, we aim to provide:

(i) A flexible phase-retrieval toolbox for expert users.

(ii) An interface, currently a command-line interface and in the future a graphical user interface, for non-expert users.

(iii) Tools for deployment on computer clusters and heterogeneous computing infrastructures.

(iv) Tools for implementation and development of phase-retrieval algorithms.

(v) High level of modularity to facilitate the integration of different packages, *e.g.* registration, tomography, fast Fourier transform (FFT), reading and writing data, and visualization.

The package consists of a number of modules, namely:

(i) *Phaseretrieval* contains functionality for phase-retrieval algorithms. Table 1 ▸ lists the currently implemented algorithms.

(ii) *Propagator* contains functionality for the propagation of a wavefield and generation of intensities. The propagators are typically used in iterative phase-retrieval algorithms. Currently, a Fresnel operator is implemented, as well as linear­ized versions based on transport of intensity (TIE) (Teague, 1983 ▸) and contrast transfer function (CTF) equations (Guigay, 1977 ▸).

(iii) *Tomography* contains functionality for tomographic operations: forward projection, back-projection, as well as 3D image processing operators. The main part of this module wraps other codes for tomographic reconstruction, currently *PyHST* (Mirone *et al.*, 2014 ▸), with a wrapper for *TomoPy* (Gürsoy *et al.*, 2014 ▸) in development.

(iv) *Dataset* contains functionality to read and write images in different formats and from different data sources. Current data sources are ESRF-style EDF data sets and NanoMAX-style HDF5 data sets, with support for TOMCAT-style TIF data sets in development. Compatibility with other data sources is planned, *e.g.* by interfacing DXChange (De Carlo *et al.*, 2014 ▸).

(v) *Utilities* contains supporting functionality, such as image registration and visualization. Registration is currently implemented as a wrapper of *Elastix* (Klein *et al.*, 2010 ▸; Shamonin *et al.*, 2014 ▸) via *pyElastix* (Klein, 2019 ▸).

(vi) *Parallelizer* contains functionality for parallelization. This is implemented as decorators decorating functions that take a range of projections as input. Currently, the supported infrastructures are OAR (http://oar.imag.fr/start), SLURM (Yoo *et al.*, 2003 ▸), and serial processing. Switching between infrastructures requires the modification of one line in the configuration file.

*PyPhase* is written in Python 3.

## 3. Phase-retrieval algorithms   

Several phase-retrieval algorithms are currently implemented in the package. On the one hand, some of the algorithms are appropriate for the edge-enhancement regime due to model limitations on relative propagation distance or the necessity of a contact-plane image. On the other hand, appropriate algorithms for the holographic regime are provided, *e.g.* algorithms where the contact plane radiograph is not necessary, capable of reconstructing the attenuation and improving the high-frequency content (necessary for high-resolution imaging). The algorithms currently implemented in *PyPhase*, suitable for both edge-enhancement and holographic regimes, are listed in Table 1 ▸.

The algorithms more appropriate for the edge-enhancement regime currently implemented are based on linearization of the relationship between the exit wave and the contrast in the detection plane, which permits efficient, filtering-based algorithms to be derived, within some restrictions on the imaging conditions. The TIE-based algorithms are based on a linearization with respect to the propagation distance and are hence naturally limited to relatively short propagation distances (in other words large Fresnel numbers). Assuming a weakly attenuating object (WTIE) or a homogeneous object (TIEHOM) permits phase retrieval from a single measurement. If multiple images are used, the least-squares solution (Yu *et al.*, 2018 ▸) is used. The TIEHOM method requires the ratio of the refractive index decrement over attenuation coefficient (δ/β) as a parameter. If multiple distances are used, a regularization parameter α can be given (Yu *et al.*, 2018 ▸). The mixed approach combines aspects of the CTF and the TIE. It permits at the same time strong attenuation and relatively long propagation distances but requires at least two images: one without propagation between sample and detector (contact or attenuation image) and one with propagation. It also assumes the object to be slowly varying, which in some cases can reduce the achievable resolution in the reconstructed image (Guigay *et al.*, 2007 ▸). The mixed approach can be used in conjunction with different image priors to regularize the low-frequency content in the retrieved image. Similarly to the TIEHOM algorithm, the mixed approach can be used with a homogeneous prior, but also with a multi-material prior and a heterogeneous object prior. The homogeneous object prior requires, like the TIEHOM, the δ/β ratio as parameter (Langer *et al.*, 2010 ▸). This parameter can be determined empirically, either by trial and error or automatically (Rositi *et al.*, 2014 ▸), or by using the theoretical value calculated with, for example, the *XOP* software (Sanchez del Rio & Dejus, 2011 ▸). The multi-material prior requires thresholds for the different materials (usually hard/soft tissue), and corresponding δ/β ratios (Langer *et al.*, 2012*a* ▸). The heterogeneous object prior requires a functional relationship between δ and β (Langer *et al.*, 2014 ▸). All three priors require a regularization parameter α.

Holographic regime algorithms listed in Table 1 ▸ can be used for this regime but also for the edge-enhancement regime. The CTF is also based on a linearization, but with respect to the variations in the wave itself. This means that the model is valid if attenuation is weak. This algorithm permits to reconstruct both attenuation and phase. It requires one or several images, although in practice usually at least three images are used. For very weakly attenuating samples, a pure phase version (*CTFPurePhase*) can be used.

The remaining three algorithms are iterative schemes that can be used to refine a reconstruction from one of the other algorithms. They find local optima of the optimized function, so initialization from zero or from noise will usually not yield satisfactory results. Gradient descent (GD) is a simple steepest descent implementation. Error reduction (ER) is an iterative constraint satisfaction scheme in which each constraint in turn is imposed on the current solution. The measured images are considered one constraint. Currently implemented object domain constraints are support and non-negativity. Since the current version is more focused on simplicity and readability, we provide no performance benchmarks.

There are many more algorithms presented in the literature than currently covered by *PyPhase*. Here, a selection of simple and tested algorithms are provided as a base for further developments. The aim, however, is to progressively include as many algorithms as possible. Planned additions include, but are not limited to, compressed-sensing approaches such as ADMM-CTF (Villanueva-Perez *et al.*, 2017 ▸), non-linear conjugate gradient-based methods, Frechet derivative-based methods (Maretzke *et al.*, 2016 ▸; Davidoiu *et al.*, 2011 ▸), 3D phase-retrieval methods (Ruhlandt & Salditt, 2016 ▸), as well as other constraint satisfaction type iterative schemes (Elser, 2003 ▸; Luke, 2004 ▸) and domain constraints for these kinds of methods. Implementing a large range of algorithms would permit a fair evaluation of different algorithms in practice. Furthermore, it would help the practitioner choose the most suitable algorithm since different algorithms seem to work well in different circumstances.

## 4. Basic operations   

The package contains classes representing the building blocks of phase-retrieval algorithms. This modularity makes it simple to swap between different implementations, *e.g.* CPU, HPC or a future GPU implementation, without substantial changes to the code. The most basic operation for phase retrieval is arguably the FFT. For flexibility, the package contains a wrapper for the FFT functions (FFT, IFFT, fftshift, ifftshift, frequency variables). In the current version, the numpy FFT is used. Interfaces to other FFT packages, such as FFTW, can then be easily implemented and used with minimal modification of the code.

Iterative phase-retrieval algorithms require the implementation of propagators. An implementation of the Fresnel transform as well as a linearized propagator based on the CTF are provided. The modular implementation makes it simple to interface with other codes and algorithms.

For the implementation of 3D phase-retrieval algorithms, it is necessary to implement the Radon transform and its inverse transformation. These tomographic operations are provided as wrappers of specialized tomographic reconstruction packages. Currently *PyHST2* can be used and a *TomoPy* interface is in development. However, the modularity of *PyPhase* makes interfacing other packages straightforward.

A limiting factor for the practical application of phase retrieval in tomography is efficient data handling. We implement this as a dataset class, with subclasses that interface different sources, which provides the basic reading and writing of images in a tomographic or near-field imaging setup. Currently, classes for ESRF-style EDF data, Tomcat-style TIF data, and NanoMAX-style HDF5 data are implemented. The modularity of the code makes it simple to add new data sources, *e.g.* by interfacing DXchange (De Carlo *et al.*, 2014 ▸). This facilitates the treatment of data from different sources using the same reconstruction parameters.

Another limiting factor for phase retrieval in tomography is the computation time. Parallelization efforts are often centered on GPU processing, neglecting the large availability of computing clusters and heterogeneous computing architectures. Since the projections in a tomographic data set are usually considered independent, parallelization over the projections is ‘embarrassingly parallel’. To leverage this, a function decorator *@Parallelize* is provided, which can be applied to all functions in the package that take a range of projections as an argument. It allows to automatically split the implicit *for* loop into appropriate chunks and farm them out on the desired number of CPUs. The infrastructure used can be switched with a centralized parameter in a configuration file, thus requiring no modification of the code to change from serial to parallel computing. Currently, supported infrastructures are serial computing, and SLURM and OAR resource and task managers. The modular implementation makes it straightforward to include other infrastructures such as processes-based parallelism on multi-core workstations.

A certain number of auxiliary functionality important for phase retrieval are implemented. Most important is image registration, which is used to align the images at one projection angle. Phase-contrast images at different propagation distances and magnifications do not contain identical contrast. This effect precludes simple correlation-based approaches in all but the most trivial cases. For this case, registration algorithms based on mutual information (Weber *et al.*, 2018 ▸) seem to be more appropriate. Image registration is implemented as a class *Registrator*. Currently, this is implemented as a wrapper for the *Elastix* software through the *PyElastix* interface. *Elastix* provides all the necessary functionality, but it might not be available on all platforms. In that case, the modularity makes it straightforward to integrate other image registration codes.

A simple interface for the visualization of the different images is provided. This is to simplify the access to images, *e.g.* corrected and non-corrected projections, for verification purposes. Furthermore, it enables plotting curves related to the reconstruction process, *e.g.* the image alignment and regularization parameter choice. The current implementation relies on the *Matplotlib* package, but more advanced visualization could be used exploiting *PyPhase* modularity.

The number of dependencies is kept as low as possible. All arrays and matrix-vector operations are implemented using NumPy (https://numpy.org/). Other dependencies are currently (i) *Elastix* for image registration, since no adequate pure Python implementation is available, and (ii) *PyHST2* for tomographic operations.

## 5. Usage example   

**Figure 2:**
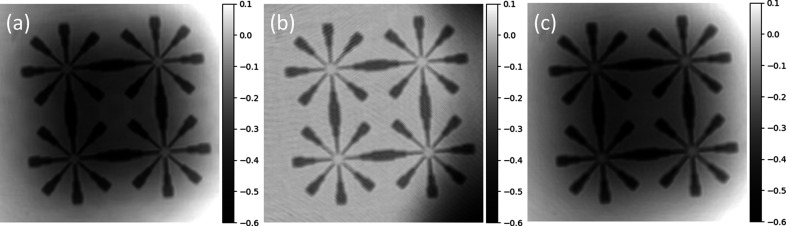
Phase-retrieved results obtained with *PyPhase* using (*a*) the CTF pure phase algorithm, (*b*) average of five iterations of successively 45 iterations of HIO, followed by five iterations of ER, separately on each acquired image, and (*c*) 20 iterations of gradient descent initialized with the CTF pure phase reconstruction. The colorbar represents the phase-reconstructed values in radians.

The implementation choices yields extremely compact code. For example, the code for the reconstruction of the image in Fig. 2 ▸(*a*) using the CTF pure phase algorithm is:[Chem scheme1] 
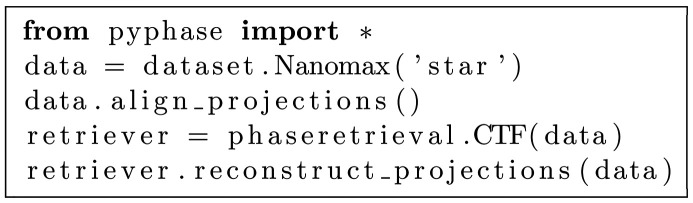


Reconstructions obtained with *PyPhase* using a similar code for three different algorithms are depicted in Fig. 2 ▸. A documentation and API is available (https://pyphase.readthedocs.io). *PyPhase* can be installed from *PyPI* using PIP.

## 6. Conclusions   

We have presented an open-source and modular Python package for phase retrieval, christened *PyPhase. PyPhase* has the potential to lower the entry barrier to phase retrieval in the Fresnel regime and thus make the technique more accessible to non-expert users. The package covers the most popular phase-retrieval algorithms presented in the literature. Furthermore, it provides tools and an interface that facilitate the implementation and deployment of other phase-retrieval algorithms. To improve usability by non-expert users, we will provide a command line interface and, in the future, a graphical user interface. Contributions are welcome: contact the authors for more information or go directly to the project repository (https://gitlab.in2p3.fr/mlanger/pyPhase).

## Figures and Tables

**Table:** 

Edge enhancement regime	Class name	Source
TIE, weak object (WTIE)	WTIE	(Bronnikov, 2002 ▸)
TIE, homogeneous objects	TIE-HOM	(Paganin *et al.*, 2002 ▸)
Mixed approach, homogeneous	MixedApproach	(Langer *et al.*, 2010 ▸)
Mixed approach, multi-material	MixedApproach	(Langer *et al.*, 2012*a* ▸)
Mixed approach, heterogeneous	MixedApproach	(Langer *et al.*, 2014 ▸)

**Table:** 

Holographic regime	Class name	Source
Contrast transfer function (CTF)	CTF	(Cloetens *et al.*, 1999 ▸)
CTF, pure-phase object	CTFPurePhase	(Cloetens *et al.*, 1996 ▸)
Gradient descent	GD	(Langer *et al.*, 2012*b* ▸)
Hybrid Input–Output (HIO)	HIO_ER	(Fienup, 1978 ▸)
Error reduction (ER)	HIO_ER	(Gerchberg & Saxton, 1972 ▸)
